# Prevalence of Hypothyroidism Among Dialysis Patients in Eastern Region, Saudi Arabia

**DOI:** 10.7759/cureus.33807

**Published:** 2023-01-15

**Authors:** Hessah A Al Hussaini, Muthana A Al Sahlawi, Fai Alhussain, Lama A Alja’afari, Hussain I Aljohar, Mohammed S Al-Ramadhan, Sayed Ibrahim Ali, Bashaeer Al Jalal, Omar Alomair, Mohammed Almulhim

**Affiliations:** 1 Internal Medicine Department, College of Medicine, King Faisal University, Al Ahsa, SAU; 2 Internal Medicine Department, College of Medicine, King Faisal University, AlAhsa, SAU; 3 Family & Community Medicine, King Faisal University, Al Ahsa, SAU

**Keywords:** prevalence, peritoneal dialysis (pd), hd (hemodialysis), end-stage renal failure, :- hypothyroidism

## Abstract

Background

Hypothyroidism carries significant morbidity among the general population and is more common among patients with reduced Glomerular filtration Rate (GFR). Patients with reduced GFR have higher cardiovascular morbidity and mortality, which might be increased in the presence of hypothyroidism. A thyroid function test is not routinely included in predialysis workups.

Aim

The aim was to explore the prevalence of hypothyroidism among hemodialysis and peritoneal dialysis patients at a single large center in Al-Ahsa, Saudi Arabia.

Methods

A chart-review cross-sectional study was conducted at Al Jabr Kidney Center from February to May 2022. It included adult patients on hemodialysis or peritoneal dialysis. Data was extracted through a pre-structured data extraction sheet to avoid data collection errors. Extracted data included the patient's demographic data, causes of renal failure, and comorbidities besides laboratory investigations and thyroid profile.

Results

A total of 99 patients were included, with their ages ranging from 15 to 89 years, with a mean age of 51.3 ± 16.9 years old. The exact 76 (76.8%) patients were males. Exact five (5.1%) patients had high thyroid stimulating hormone (TSH), nine (9.1%) had low TSH, and 85 (85.9%) were euthyroid. There was no difference in the prevalence of hypothyroidism according to the type of dialysis (p=0.872). Dialysis adequacy was achieved in the majority of included patients based on Kt/V (80.5%) and URR (61.7%) regardless of thyroid status (p=0.115 and 0.653, respectively). The presence of hypertension and erythropoietin were more prevalent among patients with high TSH levels.

Conclusion

We concluded that hypothyroidism among dialysis patients was less common in our study compared to previously reported prevalence nationally and internationally. The prevalence of hypothyroidism was similar in both hemodialysis and peritoneal dialysis patients, and it did not affect dialysis adequacy. Hypertension and erythropoietin were more common among our dialysis patients with hypothyroidism. Screening for thyroid disorders among chronic disease patients (especially on dialysis) is essential to improve the quality of care.

## Introduction

Hypothyroidism is a significant morbidity source affecting a considerable portion of the general population. The prevalence of clinical and subclinical hypothyroidism is higher in patients with reduced Glomerular Filtration Rate (GFR) than in those with preserved kidney function [1-10]. Moreover, cardiovascular disease is highly prevalent and the leading cause of mortality among end-stage renal disease (ESRD) patients, which can be exacerbated by hypothyroidism [11-20]. However, many centers do not perform regular screening for hypothyroidism in dialysis patients, with thyroid stimulating hormone (TSH) not part of the initial blood work for new dialysis patients. This could result in undiagnosed hypothyroid status in some of these patients. Coupled with the fact that hypothyroidism might be overlooked as some of its common symptoms could be mistakenly attributed to uremia or anemia [21].

This study aimed to explore the prevalence of hypothyroidism among hemodialysis (HD) and peritoneal dialysis (PD) patients at a single large center in Al-Ahsa, Saudi Arabia. We also aimed to identify the association between hypothyroidism and dialysis adequacy.

## Materials and methods

A chart review cross-sectional study was done for adult patients undergoing dialysis and followed in Al Jabr Kidney Center, the largest dialysis center in the Eastern region of Saudi Arabia [8]. The relevant parameters of all included patients will be collected: patients' demographics, chronic illnesses, use of thyroid medication, cause of chronic kidney disease, type of dialysis, anthropometric measurements, and routine monthly laboratory testing results. Thyroid function tests were checked, in included patients that were done for dialysis monthly laboratory testing. Patients with or without existing thyroid disease were included.

A sample size of 81 patients was considered a representative sample of all dialysis patients in Saudi Arabia. It was calculated based on the following formula: n=p(1-p)z^2^/e^2^, where p = prevalence of dialysis patients in Saudi Arabia which is 0.056% according to the total dialysis patients of 19,715 patients in Saudi Arabia in the year 2020 according to Saudi Center of Organ Transplantation [22] and Saudi population of 35,013,414 in the year 2020 according to General Authority for Statistics - Kingdom of Saudi Arabia [23-24], z = 1.96 for a confidence level (α) of 95% and e = margin of error of 0.05. The sample was selected using a convenience sampling technique.

Hypothyroidism was defined as TSH higher than reference ranges or if the patient has a background history of hypothyroidism under replacement.

After data were extracted, it was revised, coded, and fed to statistical software IBM SPSS version 22 (SPSS, Inc. Chicago, IL). All statistical analysis was done using the chi-square test, and a p-value less than 0.05 was statistically significant. Descriptive analysis based on frequency and percent distribution was done for all variables, including the patient's bio-demographic data. However, causes of renal failure, comorbidities, and frequency of hypothyroidism were graphed. Also, laboratory investigation was displayed using mean with standard deviation. Crosstabulation for relations was used for all these relations using Persons' chi-square test and exact probability test for small frequency distributions.

## Results

A total of 99 patients were included, with their ages ranging from 15 to 89 years, with a mean age of 51.3 ± 16.9 years old. The exact 76 (76.8%) patients were males. A total of 96 (97%) were Saudi. As for body mass index (BMI), 44 (44.4%) had normal body weight, 18 (18.2%) were overweight, and 37 (37.4%) had obesity. A total of 82 (82.8%) were on hemodialysis (HD), and 17 (17.2%) were on peritoneal dialysis (PD). Dialysis adequacy was calculated based on the Kt/V and urea reduction rate (URR) and was adequate in our patients (table 1).

**Table 1:** Bio-demographic data of dialysis patients (N=99)

Bio-demographic data	N	%
Age in years		
< 40	29	29.3%
40-60	38	38.4%
> 60	32	32.3%
Gender		
Male	76	76.8%
Female	23	23.2%
Nationality		
Saudi	96	97.0%
Non-Saudi	3	3.0%
BMI		
Normal weight	44	44.4%
Overweight	18	18.2%
Obesity	37	37.4%
Type of Dialysis		
Hemodialysis	82	82.8%
Peritoneal dialysis	17	17.2%
Dialysis Adequacy	Mean	SD
Duration of Dialysis in Months	45.16	47.19
Kt/V (≥1.2)	1.3	0.19
Urea Reduction Rate URR (≥65%)	67.11	7.52

**Figure 1:**
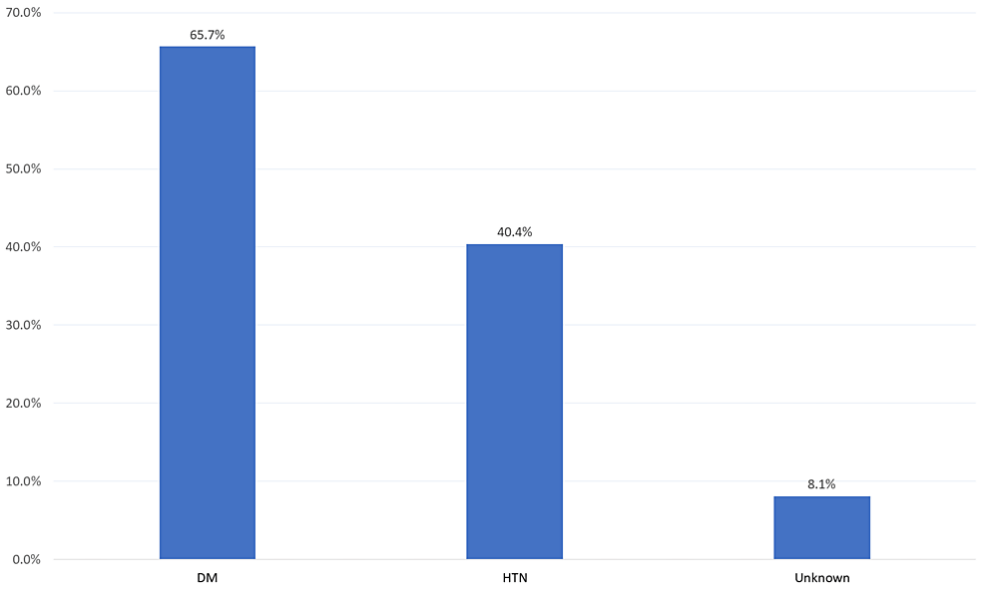
Causes of renal failure among dialysis patients

The most reported causes of renal failure were Diabetes Mellitus (DM) (65.7%), followed by Hypertension (HTN) (40.4%). The cause was unknown among 8.1% of the patients, and 12% of patients had variable causes of renal failure, including, but not limited to, glomerulonephritis, reflux nephropathy, and polycystic kidney disease (Figure 1).

**Figure 2:**
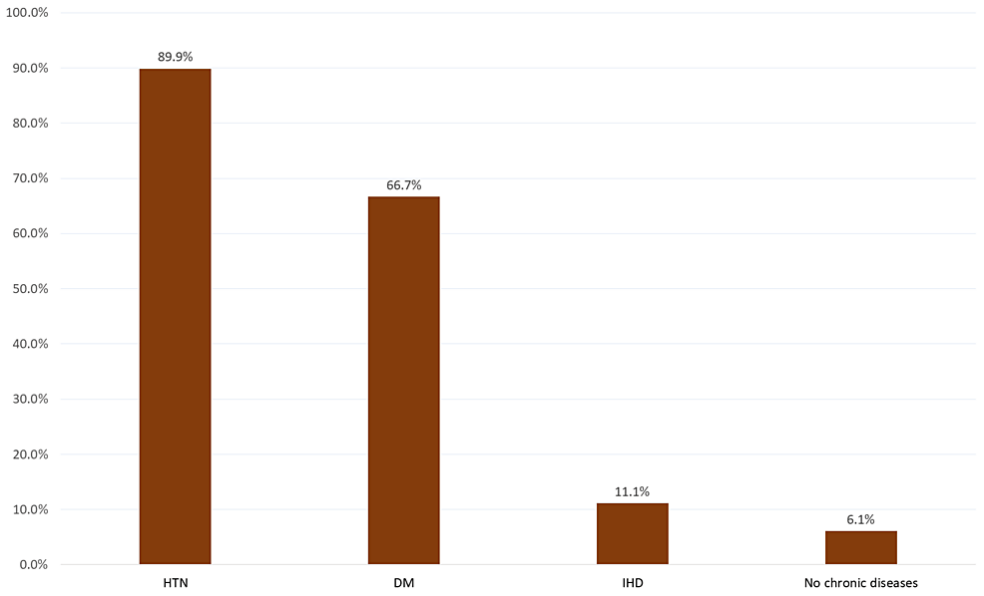
Co-morbidities among dialysis patients

Hypertension was the most reported comorbidity (89.9%), followed by DM (66.7%), ischemic heart disease (IHD) (11.1%), and CVA (4%), while 6.1% had no chronic health problems other than chronic kidney disease (Figure 2).

**Figure 3:**
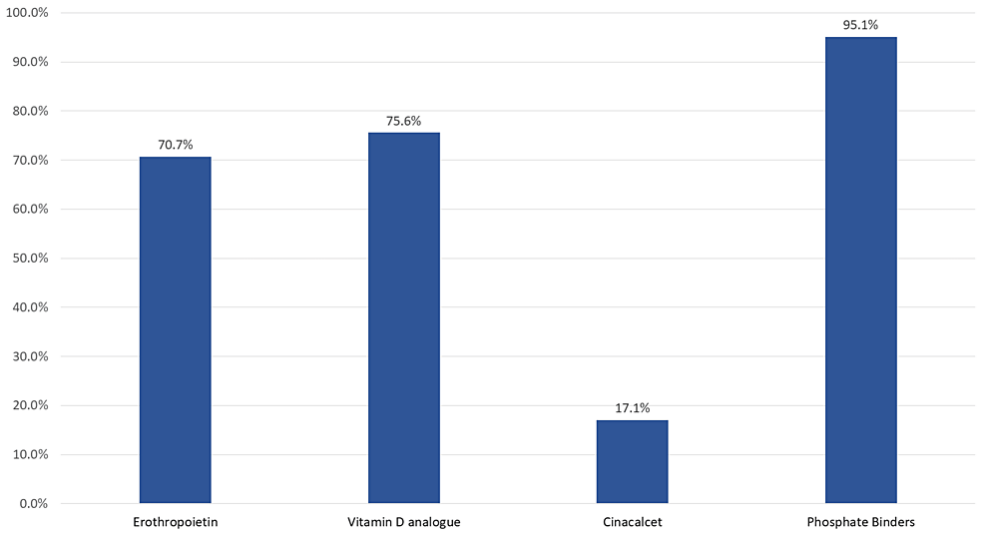
Medications used in dialysis patients

Regarding the medications used in dialysis patients, 95.1% were on phosphate binders, 75.6% were on vitamin D analogue, 70.7% were on erythropoietin, and 17.1% were on erythropoietin were on Cinacalcet. We had only one patient using levothyroxine, as the patient had existing hypothyroidism (Figure 3).

Most of our patients achieved within-target laboratory investigations, including hemoglobin, potassium, calcium, parathyroid hormone (PTH), and hemoglobin A1c (Table 2).

**Table 2:** Laboratory investigations among dialysis patients PTH: parathyroid hormone

Laboratory investigations	Mean	SD
Hemoglobin (10-12 g/dL)	10.33	1.65
Phosphate (0.9-1.4 mmol/L)	1.59	.64
Potassium (3.5-5.1 mmol/L)	4.77	.97
Albumin (35-50 g/L)	33.7	5.4
Corrected Ca (2-2.4 mmol/L)	2.15	.19
PTH (10-52.5 pmol/L)	47.8	39.3
HbA1C in patients with DM only (<8%)	6.6	1.8

**Figure 4:**
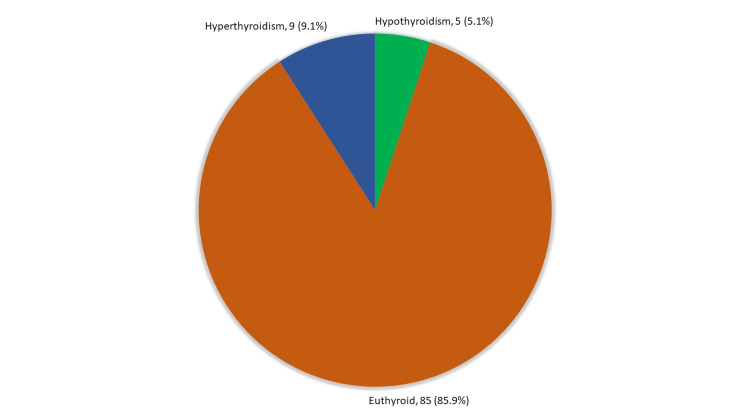
Thyroid profile among dialysis patients

Exact of the five (5.1%) patients had high TSH, nine (9.1%) had low TSH, and 85 (85.9%) were euthyroid. The TSH level ranged from 0.15-7.16 μIU/L with a mean value of 2.38 ± 1.46 μIU/L (Figure 4).

We compared all relevant data collected with different TSH levels to find any possible association. We found no association between TSH level and gender, BMI, dialysis type, and dialysis adequacy (p >0.05). Hypertension was the only comorbidity present in hypothyroid patients (p=0.05). Among the medications used in dialysis patients, erythropoietin was used more in hypothyroid patients, and vitamin D analogue was used less in hypothyroid and hyperthyroid patients (Table 3).

**Table 3:** Association between TSH levels among different variables (Gender, BMI, Dialysis Type, Dialysis Adequacy, Comorbidities, and Medication). *Statistically significant. URR: Urea reduction ratio; DM: Diabetes mellitus; HTN: hypertension; IHD: Ischemic heart disease

Factors	Thyroid profile						
	Hyperthyroidism		Euthyroid		Hypothyroidism		p-value
	N	%	N	%	N	%	
Gender							0.748
Male	4	5.3%	66	86.8%	6	7.9%	
Female	1	4.3%	19	82.6%	3	13.0%	
BMI							0.185
Underweight	2	25.0%	5	62.5%	1	12.5%	
Normal	1	2.8%	31	86.1%	4	11.1%	
Overweight	0	0.0%	17	94.4%	1	5.6%	
Obesity	2	5.4%	32	86.5%	3	8.1%	
Dialysis Modality							0.872
Hemodialysis	4	4.9%	70	85.4%	8	9.8%	
Peritoneal Dialysis	1	5.9%	15	88.2%	1	5.9%	
Dialysis Adequacy							
Kt/V (≥1.2)	2	3.0%	56	84.8%	8	12.1%	0.115
URR (≥65%)	2	4.0%	42	84.0%	6	12.0%	0.653
Comorbidities							
DM	2	3.0%	57	86.4%	7	10.6%	0.349
HTN	3	3.4%	77	86.5%	9	10.1%	0.050*
IHD	2	18.2%	9	81.8%	0	0.0%	0.068
Heart Failure	0	0.0%	2	100.0%	0	0.0%	0.845
Atrial Fibrillation	0	0.0%	2	100.0%	0	0.0%	0.845
Stroke	0	0.0%	4	100.0%	0	0.0%	0.709
Anemia	1	2.4%	37	88.1%	4	9.5%	0.365
Medications							
Erythropoietin	1	1.7%	49	84.5%	8	13.8%	0.025*
Vitamin D analogue	1	1.6%	56	90.3%	5	8.1%	0.030*
Cinacalcet	1	7.1%	13	92.9%	0	0.0%	0.380
Phosphate Binders	4	5.1%	66	84.6%	8	10.3%	0.697

## Discussion

Hypothyroidism prevalence has been studied among patients with chronic kidney disease. It has been linked to anemia, volume overload, cardiovascular morbidity, and mortality among chronic kidney disease patients compared to other patients with normal thyroid function [1-3,6,9-12,15,20-21]. Nevertheless, hypothyroidism among dialysis patients has been studied and showed similar observations [4,7,10,13-14,16,19].

To our knowledge, this is the first study about the prevalence of hypothyroidism among dialysis patients in this region of Saudi Arabia. We discovered five patients with hypothyroidism in our included patients. Hypothyroidism was not related to patient age, gender, BMI, cause of renal failure, or type of dialysis. Hypothyroidism in our study was reported less than in the Alshammari et al. study done in Security Force Hospital in Saudi Arabia. They reported that 17.66% of dialysis patients had hypothyroidism [3]. It was also less than what Rhee et al. reported in the study of hypothyroidism among dialysis patients, which was 12.9% [13].

Interestingly, the adequacy of dialysis was not affected by any abnormality in thyroid function. This was different from other international studies where the adequacy of dialysis was affected by the presence of hypothyroidism [10,13-14,16].

It is well known that undetected and untreated hypothyroidism can lead to significant morbidity and mortality. In addition, cardiovascular morbidity and mortality are higher among dialysis patients, and hypothyroidism can worsen their condition [13-20]. We found that hypertension is more common among patients with hypothyroidism but not other cardiovascular diseases commonly seen among patients with thyroid disorders (for ex, ischemic heart disease, heart failure, and atrial fibrillation).

Anemia is common among dialysis patients, and patients frequently need erythropoietin to maintain hemoglobin levels within the target [20-21]. Although our patient's hemoglobin level was not affected by hypothyroidism, hypothyroid patients tend to use erythropoietin more than euthyroid patients. This indirectly means that hypothyroid patients need more anemia management. Erythropoietin use is known to cause elevated blood pressure, which may be one reason our patients with hypothyroidism had more hypertension than euthyroid patients.

In addition to hypothyroidism, we found nine patients with hyperthyroidism in our included patients. This was surprisingly higher than expected, yet, it was not related to patient age, gender, BMI, cause of renal failure, or type of dialysis. It did not affect dialysis adequacy.

Our study has multiple strengths. It was done in the largest dialysis center in the Eastern region and included a representative sample of the Saudi population (not only the Eastern region). All the parameters necessary to assess dialysis adequacy were measured and compared to relevant information regarding the patient's medical background and thyroid function. As a limitation, our study did not include the full thyroid function test and was based on TSH level alone. Full thyroid function will help categorize patients into clinical or subclinical thyroid disorders, thus having more accurate information about dialysis parameters [4-5,7-9]. In addition, we discovered a relatively small number of patients with thyroid disorders. Thus, we suggest a larger multi-center prospective study to include more patients with thyroid disorders and have more accurate information to compare with other national and international studies.

## Conclusions

The current study showed that hypothyroidism was reported in lower frequency among dialysis patients than in the national and international ranges. However, a larger-scale study may be needed to confirm it. The prevalence of hypothyroidism was not affected by the type of dialysis. Thyroid status did not affect dialysis adequacy, but hypertension and the use of erythropoietin were more prevalent among hypothyroid patients. We recommend routinely screening for thyroid diseases in all chronic kidney patients, with particular attention to dialysis patients.
